# What We Gain From Machine Learning Studies in Headache Patients

**DOI:** 10.3389/fneur.2020.00221

**Published:** 2020-04-09

**Authors:** Roberta Messina, Massimo Filippi

**Affiliations:** ^1^Neuroimaging Research Unit, Division of Neuroscience, Institute of Experimental Neurology, Milan, Italy; ^2^Neurology Unit, IRCCS San Raffaele Scientific Institute, Milan, Italy; ^3^Vita-Salute San Raffaele University, Milan, Italy

**Keywords:** headache, neuroimaging, machine learning, migraine, biomarkers, cluster headache

Primary headache disorders, such as migraine and cluster headache, are among the most prevalent and debilitating neurological diseases worldwide (1). An increasing recognition of the importance of these diseases has led to a growing interest in understanding their pathophysiology and developing new treatments. From the once popular *Vascular* theory that described primary headaches as vascular disorders, the field has now moved to the *Neuronal* theories involving either the peripheral or central nervous system, or both (2). It is now recognized that primary headaches are not simply a disease of recurrent pain attacks but a complex and multifaceted brain disorder. There is evidence that in predisposed headache patients various cortical, subcortical, and brainstem regions are activated, and key neuropeptides are released during the headache attack (3). Neuroimaging techniques have made a tremendous contribution to our understanding of headache pathophysiology, providing insights into human brain networks that might account for the pain and the broad symptomatology characterizing the headache attacks. The brainstem, including the trigeminovascular pathway, thalamus and hypothalamus seem to have a pivotal role in triggering the migraine and cluster headache attacks. Widespread structural and functional alterations in multisensory processing brain areas have also been shown in both conditions during the interictal and ictal phase (4).

A better understanding of the mechanisms responsible for the generation of the headache attacks allows the identification of novel therapeutic targets. In conjunction with progress in theories of the pathophysiology of primary headaches, the understanding of the mechanisms of action of acute and preventive treatments for migraine and cluster headache has evolved. A few neuroimaging studies have explored the therapeutic effects of pharmacological and non-pharmacological therapeutic approaches commonly used against migraines and cluster headaches, suggesting a potential central mechanism of action of these therapies (5–7).

Although much progress has been made in the understanding of migraine and cluster headache, there are still many unsolved questions to address. Many studies suggested that brain alterations in headache patients might change dynamically over time, since they differ according to the headache phase, frequency of attacks, and disease duration (8, 9). However, some brain alterations are not influenced by the disease activity, suggesting that they might represent brain biomarkers that predispose to the disease (10, 11). Further unanswered questions are whether it is possible to identify a specific neuroimaging pattern for each different headache phenotype and if alterations in the function and structure of nociceptive brain areas are headache-specific or common to other chronic pain disorders. Moreover, imaging biomarkers that could predict treatment response of headache patients are scarce.

A valuable strategy to reduce the unmet needs in the understanding of primary headaches is to study headache patients using machine learning approaches. These methods have been employed to study patients with neurological or psychiatric conditions—like Alzheimer's disease, depression, and chronic pain disorders—in order to identify neuroimaging biomarkers, which could be used to predict clinical outcomes, including diagnostic categories, measures of symptoms, prediction of disease evolution, and treatment response (12, 13). There are two main machine learning approaches: supervised and unsupervised. Supervised machine learning algorithms are trained to automatically classify individuals into predefined groups, e.g., patients or healthy controls, and yield an associated accuracy indicative of how well the model could generalize to future individual cases (12, 14). At a more detailed level, a machine learning classifier is a function that takes the values of various features (e.g., different imaging patterns) in an example and predicts the class that example belongs to (e.g., patient or control). The goal is to develop a “classifier” that identifies the relation between each example and its respective category with high accuracy (15). Based on what the algorithm has learned, it will be then able to classify new, previously unseen data to one of the predefined categories (12). By contrast, unsupervised machine learning models are data-driven automated approaches that, without the availability of a priori information supplied by the operator, seek to classify uncategorized data, with the primary aim of discovering unknown, but potentially useful information in the data (15). These classification models include a “training” phase in which training data are used to develop an algorithm able to discriminate between groups, and a “testing” phase in which the algorithm is used to blind-predict the group to which a new observation belongs.

The main advantages of using machine learning approaches are that they allow inference on an individual patient basis and are sensitive to subtle and spatially distributed patterns of disease-induced changes in the brain that might be undetectable at group level comparisons (12, 16). The evaluation of the performance of the model in a new subset of individuals provides valid estimates of how well the discriminative model generalizes to new data, enhancing the clinical significance of these approaches (16).

Recent machine learning studies have focused on the diagnosis of migraine. Machine learning algorithms based on brain resting state functional magnetic resonance imaging (MRI), or morphometric MRI data have been used to identify brain signatures that discriminate migraine patients from controls (17–19). The functional connectivity of brain regions involved with processing the affective components of pain, like the insula, amygdala, temporal, and frontal lobes, discriminated migraine patients from controls with an accuracy rate of 86%. The discrimination between patients with longer (>14 years) and shorter (≤ 14 years) disease duration achieved the highest accuracy, suggesting that disease burden might influence functional reorganization in the brain. The altered patterns of functional connections that distinguish migraine patients from controls could represent migraine biomarkers that are further reinforced by recurrent pain (17). On the other hand, an unsupervised machine learning approach was not able to clearly separate migraineurs from healthy controls based upon brain morphometric measures (19). An improvement in classification performance in migraine identification can be achieved integrating functional and structural imaging metrics that disclose complementary information regarding the underlying biological processes (20).

A common objection to these studies is that the diagnosis of migraine is mainly based on taking a good clinical history. However, machine learning studies could be used to discriminate those headache patients who have challenging clinical presentations, such as patients with chronic migraine vs. patients with hemicrania continua, patients with probable migraine vs. tension type-headache or patients with episodic cluster headache vs. patients with paroxysmal hemicrania. Moreover, given the high prevalence of migraine, being sure that a control does not harbor migraine biology is very challenging. In the future, the identification of migraine-specific imaging patterns might improve the accuracy of the clinical criteria currently used for the diagnosis of migraine.

Supervised and unsupervised machine learning approaches have been applied for migraine patient stratification. Classifiers containing MRI measures of brain cortical thickness, cortical surface area, and regional volumes of areas involved in nociception accurately classified individuals as having chronic migraine, achieving an accuracy of 84% when compared to episodic migraine patients (18). A data-driven classification study identified two subgroups of migraine patients based upon their brain structures, with one subgroup having longer disease duration, higher migraine-related disability and more severe allodynia symptoms during migraine attacks. Thus, highlighting the role of machine learning models in identifying migraine patients with different disease courses (19). Future machine learning studies combining clinical, structural, and functional imaging measures will be valuable for identifying episodic migraine patients who are at risk of evolving into a chronic form. These models could provide a basis for early intervention, which can potentially prevent or even reverse the course of the disease. In the future, the study of patients with different types of headache and patients with other chronic pain disorders using machine learning techniques might provide brain “signatures” that are specific for the different conditions. Thus, providing important information about the relationships between disorders and symptoms at the biological level.

One of the most promising applications of machine learning techniques lies in their aid in customizing patients' treatment based on imaging brain fingerprints. Previous studies have shown the ability of machine learning approaches to predict treatment response in patients with major depression based on functional and structural brain imaging patterns (21, 22). It is desirable that future use of machine learning techniques, imaging, and clinical data, would allow us to identify objective biomarkers that might facilitate the selection of the most appropriate treatment for each headache patient. Objective biomarkers that predict treatment response can improve headache patients' management and reduce unmet treatment needs. Optimized treatments tailored to the individual patient are essential to improve headache patients' quality of life and increase patients' productivity.

Despite increasing interest in these emerging techniques, many challenges remain to be solved. None of the machine learning studies in headache patients have validated the accuracy of their models in independent datasets, so far. Moreover, the generalizability of the classification models across different sites and scanners should be evaluated. Large-scale datasets of headache patients are also needed.

## Author Contributions

RM and MF have both contributed to the study concept and drafting and revising the manuscript.

### Conflict of Interest

The authors declare that the research was conducted in the absence of any commercial or financial relationships that could be construed as a potential conflict of interest.
